# Prevalence and Determinants of Undernutrition in Schoolchildren in the Kilombero District, South-Eastern Tanzania

**DOI:** 10.3390/tropicalmed9050096

**Published:** 2024-04-25

**Authors:** Elihaika G. Minja, Emmanuel C. Mrimi, Winfrida P. Mponzi, Getrud J. Mollel, Christin Lang, Johanna Beckmann, Markus Gerber, Uwe Pühse, Kurt Z. Long, Honorati Masanja, Fredros O. Okumu, Marceline F. Finda, Jürg Utzinger

**Affiliations:** 1 Environmental Health and Ecological Sciences, Ifakara Health Institute, Ifakara P.O. Box 53, Tanzania; emrimi@ihi.or.tz (E.C.M.); wmponzi@ihi.or.tz (W.P.M.); gjoseph@ihi.or.tz (G.J.M.); hmasanja@ihi.or.tz (H.M.); fredros@ihi.or.tz (F.O.O.); lfinda@ihi.or.tz (M.F.F.); 2 Swiss Tropical and Public Health Institute, Kreuzstrasse 2, CH-4123 Allschwil, Switzerland; kurt.long@swisstph.ch (K.Z.L.); juerg.utzinger@swisstph.ch (J.U.); 3 Faculty of Medicine, University of Basel, Petersplatz 1, CH-4003 Basel, Switzerland; 4 Department of Pediatrics and Child Health, Muhimbili University of Health and Allied Sciences, Dar es Salaam P.O. Box 65001, Tanzania; 5 Department of Sport, Exercise and Health, University of Basel, Grosse Allee 6, CH-4052 Basel, Switzerland; christin.lang@unibas.ch (C.L.); johanna.beckmann@unibas.ch (J.B.); markus.gerber@unibas.ch (M.G.); uwe.puehse@unibas.ch (U.P.); 6 School of Biodiversity, One Health and Veterinary Medicine, University of Glasgow, Glasgow G12 8QQ, UK; 7 School of Life Science and Bioengineering, Nelson Mandela African Institution of Science & Technology, Arusha P.O. Box 447, Tanzania

**Keywords:** anaemia, malaria, nutritional status, schoolchildren, Tanzania, undernutrition

## Abstract

Childhood undernutrition is a major issue in low- and middle-income countries, affecting the health, well-being, and educational outcomes of schoolchildren. This study aimed to assess the prevalence and associated factors of stunting, wasting, and underweight among schoolchildren in peri-urban areas in the south-eastern part of Tanzania. A cross-sectional study was conducted involving 930 children aged 6–12 years from four primary schools from July to August 2019. The WHO Anthro Survey Analyzer was employed to estimate the prevalence of stunting, wasting, and underweight, while logistic regression analyses examined sociodemographic background, malaria infection, anaemia, anthropometric measures, and dietary diversity score as potential factors. The prevalence of stunting, wasting, underweight, overweight, and obesity was 11.8%, 4.3%, 3.9%, 11.1%, and 2.0%, respectively. Overall, 1.5% of the children had malaria, as determined by rapid diagnostic tests, and 0.4% had severe anaemia. Univariate analysis indicated higher odds of undernutrition among children aged 9–12 compared to their younger peers. Stunting was more common among children with low and medium dietary diversity. Anaemia was found in 11.2% of schoolchildren, and severe anaemia was associated with wasting. Multivariate analysis revealed that age and low dietary diversity were significantly associated with undernutrition. These findings emphasise the need for school-based health and nutrition programmes targeting children beyond the age of 5 to improve their nutritional status and mitigate potential adverse effects on health, cognition, and academic achievement. Regular assessment of the nutritional status of schoolchildren is warranted.

## 1. Introduction

Malnutrition, which comprises undernutrition and overnutrition, is a major health issue among children, particularly in low- and middle-income countries (LMICs) [1]. In 2022, globally, 148.1 million children under the age of 5 were stunted, 45 million wasted, and 38.7 million overweight [1]. In spite of the global decline in stunting in children under 5 years of age, the total number of stunted children in Africa has increased since 2000, from 54.4 million to 61.4 million, with the number expected to reach 61 million by 2025 [1,2].

Undernutrition, obesity/overweight, anaemia, malaria, and multiple micronutrient deficiencies are common health problems faced by school-aged children in sub-Saharan Africa [3,4,5,6,7]. The 2021 School Malaria and Nutrition Survey (SMNS) report indicated that 11.8% of children aged 5–16 suffered from malaria; 32% were anaemic [8]; 25.0% were stunted; 11.7% were underweight; 11.0% were thin, and 5% were overweight or obese [8]. Malnutrition often begins in the pre-school period and may progress to school age. If left untreated, it can significantly harm schoolchildren’s academic performance, cognitive development and intelligence quotient (IQ). Delayed school enrolment, increased absenteeism, early dropout and poor academic performance in school contribute to diminished well-being during childhood and have long-lasting consequences. These adverse effects can result in lower educational achievement, lower muscular strength and reduced work capacity in adulthood [9,10,11]. Overweight or obese school-aged children are at an elevated risk of high blood pressure, metabolic syndrome, type 2 diabetes, and psychological disorders [12]. On the other hand, when children’s environments undergo positive changes, such as enhanced access to nutritious food, healthcare, and education, they may experience improvements in their physical, cognitive, and socio-emotional development [13,14,15]. Hence, it is crucial to prioritize comprehensive nutritional assessments and develop effective nutritional intervention programmes within communities to determine the prevalence of undernutrition in primary schoolchildren [16]. By implementing targeted interventions during this developmental period, the long-lasting impact of undernutrition on health can be mitigated and supported in earlier years [17].

The primary focus of many researchers has been on studying malnutrition in children under the age of 5 and pregnant women, often neglecting schoolchildren from health and nutrition surveys or monitoring efforts. The purpose of this study was twofold. First, we examined the prevalence of undernutrition among primary schoolchildren. Second, we explored the association between undernutrition and underlying risk factors.

## 2. Materials and Methods

### 2.1. Study Design

This study was a school-based cross-sectional survey conducted from July to August 2019, involving primary schoolchildren aged 6–12 recruited from four public primary schools, Katindiuka, Kibaoni, Kining’ina, and Miembeni, in the south-eastern part of Tanzania. This study is part of a larger cluster-randomized, placebo-controlled trial aimed at assessing the effects of physical activity and multi-micronutrient supplementation on children’s growth, health, and well-being in three African countries, namely, Tanzania, South Africa, and Côte d’Ivoire [18]. Schoolchildren were included if they were aged between 6 and 12, had written parental or guardian consent and were not participating in any other research project. Schoolchildren whose parents/guardians did not provide written informed consent, denied oral assent, and participated in food/nutritional programmes in the past 6 months were excluded.

### 2.2. Participants and Procedures

Parents/guardians of the schoolchildren attending classes 1–4 were invited to participate in an information session that took place at school. The researchers informed the parents/guardians about the aim and objectives of this study, procedures, expected duration, benefits, and potential risks. Parents/guardians were asked to complete an informed consent form to allow their children to participate. For illiterate parents/guardians, the information sheet was read aloud or orally translated into the local language. Parents/guardians were informed that participation was voluntary, data would be treated confidentially, and participants may withdraw from the study at any time without further obligations. Then, schoolchildren whose parents provided written consent were called to a meeting where they were informed about this study’s goals, methods, benefits, and potential risks. Each schoolchild invited was asked to provide a written assent.

### 2.3. Study Area

This study was conducted in four public primary schools in Ifakara, a small but rapidly growing town in the Kilombero district that lies at 8.1336 South latitude and 36.855 East longitude. The altitude ranges from 120 to 350 m above mean sea level. Ifakara town has 40 primary schools (33 public and 7 private schools). The main economic activity in the area is rice cultivation, but residents also practice fishing, forestry, and livestock keeping. A more detailed description of the study area is provided elsewhere [19].

### 2.4. Anaemia and Malaria Infection Status

Schoolchildren provided a finger-prick blood sample collected by trained laboratory technicians for the measurement of haemoglobin levels and the diagnosis of malaria. Anaemia was tested by measuring the haemoglobin concentration using HemoCue HB 301 (Ängelholm, Sweden). The readings were categorised according to age-specific cut-off values put forth by the World Health Organization (WHO) as follows: for children aged 5–11, haemoglobin of ≥11.5 g/dL was considered normal; 11.0–11.4 g/dL was considered mild anaemia; 8.0–10.9 g/dL was considered moderate anaemia, and <8.0 g/dL was considered severe anaemia [20]. Malaria was diagnosed using SD BIOLINE Malaria Ag P.F/Pan MRDT (SD Standard Diagnostic, Inc.; Suwon, Republic of Korea), and the results were recorded as either positive or negative.

### 2.5. Anthropometric Measurements

The schoolchildren were physically assessed on-site to collect anthropometric data. Body weight and height were measured using bioelectrical impedance analysis (BIA) with a wireless body composition monitor (Tanita MC-580, Tanita Corp; Tokyo, Japan) [18]. Children were asked not to eat anything in the morning before the assessment and to empty their bladders before the assessment. Schoolchildren were asked to remove all accessories and stand barefoot on the metal plates of the machine while being guided by a research assistant to ensure optimal contact according to the manufacturer’s instructions. For the height measurement, each child stood against a stadiometer with the back erect and shoulders relaxed. Height was assessed to the nearest 0.1 cm and body weight to the nearest 0.1 kg. Nutritional status was defined according to WHO standards. Height- or length-for-age (HAZ), weight-for-age (WAZ), and body mass index (BMI)-for-age (BAZ) z-scores were computed using the WHO growth reference data. Schoolchildren with HAZ, WAZ, and BAZ scores between −2.99 and −2.00 were considered to have moderate stunting, underweight, and waste, respectively, while those with −3.00 and below were severely stunted, underweight, and wasted, respectively. Schoolchildren were classified as overweight and obese using z-score cut-off points of +2 SD and +3 SD, respectively. Weight-for-age reference data were not available for older children (above 10 years of age) due to their inability to distinguish relative height and body mass during pubertal growth spurt [21]. These variables were considered as the dependent variables for statistical analysis.

### 2.6. Caregiver Questionnaire

A structured questionnaire was administered to the caregivers of every participating child to assess sociodemographic and socioeconomic characteristics, as well as dietary intake, to determine the adequacy of macro- and micronutrient intake. Using a 24-h dietary recall modified by the Food and Agriculture Organization of the United Nations (FAO), household dietary diversity was gathered. It was changed to reflect common food products and names in Tanzania and the study area [22].

### 2.7. Statistical Analysis

Descriptive and inferential statistics were calculated using the open-source statistical software R, version 3.3 [23]. The socioeconomic status (SES) was calculated using the caregiver questionnaire data using principal component analysis (PCA), as previously described by Minja et al. [19]. WHO Anthro Software was used to convert height, weight, and age measurements to HAZ, WAZ, and BAZ. These were used to classify stunting (HAZ), wasting (BAZ), and underweight (WAZ) based on the WHO 2007 growth standards for children aged ≥5 years [21,24].

Basic descriptive parameters were reported as frequencies, percentages, and means with 95% confidence intervals (CIs). Bivariate and multivariate analyses were performed to identify the determinants of stunting, wasting, and underweight. Associations between undernutrition and independent variables were determined using binary logistic regression. This statistical test was used because stunting, wasting, and underweight were coded as binary variables. All potential predictors of stunting, wasting, and underweight were considered significant at a *p*-value < 0.05 in bivariate analyses. Those parameters known to influence the health of the child and caregiver were included in the final model as independent variables. The dietary diversity score (DDS) was calculated using a 24-h qualitative recall by mothers of the meals their children had consumed using FAO guidelines. Each of the 12 food groups was counted to obtain a DDS, which was then categorized as follows: low: ≤3 (LDD); moderate: 4−6 (MDD); high: ≥7 (HDD) [22].

## 3. Results

### 3.1. Socioeconomic and Demographic Characteristics of Study Participants

Nine hundred and thirty primary schoolchildren, along with their parents or caregivers, participated in this study. The mean age of children was 8.4 years (SD 2.2 years). Notably, the majority of children aged between 6 and 8 years comprised a higher proportion of females than males. A total of 725 parents or caregivers were interviewed, with females representing more than half of the participants. Moreover, the predominant family size ranged between four and five members. Based on the caregivers’ answers regarding their incomes and available household assets, children were classified into three SES categories, which represented approximately equal portions of children from families with poor, middle, and least poor SES (Table 1).

### 3.2. Nutritional Status

Overnutrition in the form of overweight (11.1%) and obesity (2.0%) was the most common type of malnutrition diagnosed. For undernutrition, the most prevalent type was stunting (11.8%), followed by wasting (4.3%) and underweight (3.9%). Only three schoolchildren were diagnosed with severe thinness (Table 2).

### 3.3. Potential Predictors of Stunting, Wasting, and Underweight

In terms of their overall health recalled within 7 days, 25.6% reported an existing illness. Of those, the most common symptoms were coughing and blood in the stool. Twelve children tested positive for malaria, and 5.9%, 4.9%, and 0.4% had mild, moderate, and severe anaemia, respectively. Only 12% of the children regularly ate breakfast before school. More than three-quarters of the children consumed at least two meals/day (Table 3).

### 3.4. Factors Associated with Nutrition

In the univariate logistic regression analyses, older age (9–12 years) was significantly associated with a higher prevalence of stunting (odds ratio (OR) = 3.51, 95% CI: 2.27–5.42), underweight (OR = 5.17, 95% CI: 2.34–11.42), and wasting (OR = 2.40, 95% CI: 1.24–4.66). Moreover, medium (OR = 2.63, 95% CI: 1.19–5.85) and low (OR = 3.82, 95% CI: 1.57–9.29) dietary diversity and severe anaemia (OR = 13.70, 95% CI: 1.20–156.14) were significantly associated with stunting and wasting, respectively (Table 4).

In multivariate logistic regression analyses, older age was significantly associated with a higher likelihood of undernutrition. Compared to younger peers, the odds for older children with stunting was 3.48 (95% CI: 2.03–5.96), for underweight 4.03 (95% CI: 1.34–11.89), and for wasting 2.86 (95% CI: 1.16–7.09). In addition, stunting was associated with low dietary diversity (OR = 2.79, 95% CI: 1.05–7.47) (Table 5).

## 4. Discussion

This study examined the nutritional status and associated factors of schoolchildren aged 6–12 years in the Kilombero district in the south-eastern part of Tanzania. The prevalence of stunting was 8.8%, underweight 2.8%, and wasting 4.0%. In the present sample, the prevalence of stunting was lower than that in a survey conducted in Tanzania between August and October 2019 among children aged 5–19, where 25.0% of children were stunted [8]. Similarly, the prevalence found in the present study was lower than that in other studies on indigenous populations living in the Kiberege and Kikwawila wards (23.9%) [4] or in the Chamwino and Kilosa districts (28.1%) [3]. In addition, the prevalence was lower than that reported in other countries such as Ethiopia (26.2–43.1%) [25,26], Ghana (10.4%) [27], Kenya (16.6%) [28], Uganda (22.5%) [29], and Nigeria (26%) [30]. These differences might be due to inconsistencies in the risk factors in different geographical regions, SES, and dietary diversity of schoolchildren.

In both the univariate and multivariate analyses, the likelihood of experiencing stunted growth increased with age. Specifically, schoolchildren aged 9–12 exhibited a 3.48-fold higher risk of being stunted than their younger counterparts aged 6–8. This finding is in agreement with data from other countries like Ethiopia [25], Burkina Faso [31], Madagascar [32], Pakistan [33], and India [34]. This indicates that stunting in children is a result of undernutrition, which begins during pregnancy [35] and is difficult to correct once established. In addition, older schoolchildren face unique challenges as they transition from childhood to adolescence, such as increased nutritional needs during periods of intense growth, high-energy expenditure from physical activity, participation in various extracurricular activities at home and at school that may require more energy, and limited access to nutrient-rich meals. Stunting may become more likely when people age [36].

Our findings further suggest that schoolchildren with a limited diet are at a higher risk of stunted growth. This is in line with another study conducted in LMICs [37]. Dietary diversity is a good predictor of dietary quality and micronutrient density in children [38,39]. The lack of a diversified diet has been associated with poor nutritional and health outcomes in children [40]. This might result from a diet high in cereal that is monotonous and high in anti-nutrients. These anti-nutrients have the potential to interfere with the body’s absorption of utilization of minerals [41]. Additionally, such a diet may lack micronutrients, including iron, vitamin B12, folate, and other essential elements crucial for the healthy growth of children [19,42,43]. Additionally, it is conceivable that inadequate nutrient intake during infancy and childhood has a considerable effect on the linear growth of the prescribed height at the relevant age [44].

In the present study, underweight schoolchildren accounted for 2.8% of the sample. This rate is lower than that found in a similar sample of children from the Morogoro region (3.9%) [45] in the Kiberege and Kikwawila wards (12.6%) [4] and a study conducted in Tanzania between August and October 2019, where 11.7% of children aged 5–19 were underweight [8]. Considerably higher percentages of underweight children were found in Western Kenya (7.8%) [46], Karve district in Nepal (30.9%) [47], and South India (35.9%) [48]. The differences in the prevalence of underweight might be due to environmental and geographical variations, differences in the study period, age of the study population, children from different wards, differences in the SES of the family that determine the meal frequency as well as the diversity of diet and different awareness about nutrition.

In our study, children aged 6–8 were less likely to be underweight than their older counterparts. This result is supported by other studies conducted in Ethiopia [25,49], India [34], and Nairobi [50], which revealed that nine-year-old children were more likely to be underweight. As children grew older, the odds of being underweight increased (Table 4). This might be due to the fact that the majority of schoolchildren skip meals due to food shortages in the family and that the children spend their after-school hours on household chores such as sweeping, dishwashing, and fetching water, all of which require a considerable amount of energy and nutrients. As a result, older schoolchildren might become more deficient in the essential nutrients necessary for the growth of their bodies.

In the present study, the prevalence of wasting was 4.0%, which was higher than reported in studies conducted in Nigeria (1.4%) [30], India (2%) [51], and Ecuador (2.1%) [52]. However, the prevalence found in our sample was lower than that observed in children living in Uganda (18.5%) [29], Guinea Bissau (13.8%) [53], and Burkina Faso (11.2%) [31]. In our study, the age of the schoolchildren was associated with wasting. This observation is in line with other studies in Ethiopia [54,55], Pakistan [33], and India. Wasting is associated with seasonal climatic changes (which cause food shortages and food scarcity) [31] and recent illnesses [56]. The results of our study may be due to a lack of access to sufficient food, a lack of dietary diversity or recurrent infections. In addition, most of the caregivers practized farming as their main economic activity, and the fact that the data assessment was carried out during the rice-harvesting season could be the reason for the observed results.

Furthermore, in the univariate analyses, wasted schoolchildren were more likely to be anaemic than their non-wasted peers (Table 4). This result is in line with studies conducted in Ethiopia [57,58] and elsewhere in Sub-Saharan Africa [59]. In these regions, inadequate nutritional intakes, such as iron, folate, and vitamin B12 [60], and communicable diseases, such as malaria, hookworm infection, human immune virus (HIV), and tuberculosis, are the most common causes of childhood anaemia [61]. These nutrients are essential for the growth and development of healthy red blood cells, which can predispose children to be concurrently affected by malnutrition [20,57]. Children with calorie deficiencies are more likely to have deficiencies in other micronutrients, such as iron, which is crucial for the production of haemoglobin [58].

The prevalence of overweight/obesity was 13.1% in our sample of primary schoolchildren, with 11.1% being overweight and 2.0% obese. Nevertheless, the prevalence of overweight/obesity was lower than that reported in other studies conducted in Morogoro (19%) [45], Dar es Salaam (15.9%) [62], Kilimanjaro (15%) [63], Kenya (21%) [64], and South Africa (15%) [65]. Some of these studies were conducted in large cities, which are associated with urbanization and relatively sedentary lifestyles. In contrast to what is seen in Ifakara and nearby rural areas, we expected that differences in culture, lifestyle, and other environmental factors, such as the high SES of the parents, the presence of food outlets, and the use of motorized transport, might account for the higher prevalence. Schoolchildren from rural and public schools in Tanzania are typically free to move around, play, walk to school, and regularly partake in physical activities such as gardening, sweeping/mopping the classrooms, and other activities that aid in the maintenance of a healthy weight.

Malaria prevalence measured with the mRDT was much lower in this study (1.4%) than in the SMNS-2021 report (11.8%) [8]. However, this finding is consistent with a recent entomological study that examined malaria transmission in the same wards and found that it has dramatically declined over the previous three decades [66]. Numerous factors, such as the widespread use of insecticide-treated nets (ITNs) [67], urbanization, and improved living conditions, have contributed to these decreases [68].

Anaemia was less common among schoolchildren than in the SMNS-2021 report (32%) [8], Tanga (79.6%) [68], Kikwawila and Kibaoni wards (14%) [4], and Morogoro municipality (10.1%) [69]. The low consumption of animal protein sources such as meat and other foods rich in iron and micronutrients may have contributed to the observed prevalence of anaemia [19]. Additionally, the low incidence of malaria in our study may have contributed to the low prevalence of anaemia. *Plasmodium* infection causes anaemia via the direct destruction of red blood cells in the spleen and impaired production of red blood cells in the bone marrow [70]. Additionally, the low prevalence of anaemia can be explained by the low prevalence of soil-transmitted helminths (STHs) due to ongoing deworming campaigns with albendazole against STHs and ivermectin plus albendazole against lymphatic filariasis [71]. The programme for the control and elimination of lymphatic filariasis was introduced in 2004 and for STHs in schoolchildren in 2009 [72].

One of the main strengths of this study is that it was conducted with children from an age group that may benefit from targeted interventions. The assessment of multiple factors such as caregivers’ education, participants’ age and sex, SES, household size, and dietary diversity that have the potential to influence stunting, underweight, and wasting was seen as an additional strength. Despite its unique contributions, this study has a number of limitations that deserve to be mentioned. First, this study was conducted in only four schools, which might underestimate or overestimate the “true” prevalence of stunting, underweight, wasting, overweight, and obesity in the study area. Second, this study was conducted exclusively in a peri-urban area, which limits its generalizability to rural or urban settings, as well as to the overall situation in Tanzania. Despite the random selection of schools, the results were only representative of one town. Third, a cross-sectional design was used; therefore, we could not establish a causal relationship between the predictors and outcome variables.

## 5. Conclusions

Undernutrition and obesity coexist in schoolchildren living in Ifakara, Tanzania. Efforts to reduce the negative effects of undernutrition and overweight should be incorporated into any public health strategy that targets their co-occurrence. In school settings during this critical age, interventions like encouraging physical activity and a nutritious diet may have an impact on adolescents’ nutritional results. These interventions may also help LMICs like Tanzania tackle the dual burden of disease. Furthermore, larger research initiatives or nationwide nutritional surveys are necessary to regularly evaluate the nutritional status of schoolchildren.

## Figures and Tables

**Table 1:** Sociodemographic and socioeconomic characteristics of the study participants (schoolchildren and their parents/guardians) in Ifakara, Tanzania, in 2019.

	Characteristic	Category	Frequency	Percentage (%)
Children	Age	6–8 years	516	55.5
		9–12 years	414	44.5
	Sex	Male	429	46.1
		Female	501	53.9
	School grade	1	197	21.2
		2	194	20.9
		3	247	26.6
		4	292	31.4
Parents/caregivers	Age	18–35 years	371	51.2
		36–45 years	203	28.0
		>45 years	151	20.8
	Sex	Male	180	24.8
		Female	545	75.2
	Education	No formal education	56	7.7
		Primary	597	82.3
		Secondary	68	9.4
		Tertiary	4	0.6
	Marital status	Single	154	21.2
		Married	478	65.9
		Divorced/separated	62	8.6
		Widow/widower	31	4.3
	Family size	2–3 people	107	11.6
		4–5 people	441	47.7
		≥6 people	376	40.7
	SES	Poor	221	30.5
		Middle	246	33.9
		Least poor	258	35.6

SES = socioeconomic status.

**Table 2:** Children’s nutrition status from four schools in Ifakara, Tanzania, in 2019.

Variable	Category	Frequency	Percentage
Stunting	Normal	820	88.2
	Mildly stunted	82	8.8
	Moderately stunted	28	3.0
	Severely stunted	0	0
Underweight	Normal	682	96.1
	Underweight	20	2.8
	Moderate underweight	8	1.1
	Severe underweight	0	0
Wasting	Normal	768	82.6
	Thinness	37	4.0
	Moderate thinness	0	0
	Severe thinness	3	0.3
Overnutrition	Overweight	103	11.1
	Obesity	19	2.0

**Table 3:** Malaria, anaemia, and dietary habits of schoolchildren in Ifakara, Tanzania in 2019.

Variable	Category	Frequency	Percentage
Malaria	Positive	12	1.5
	Negative	791	98.5
Body temperature	≤37 °C	756	93.8
	>37 °C	50	6.2
Anaemia	Normal	712	88.9
	Mild	47	5.9
	Moderate	39	4.9
	Severe	3	0.4
Existing disease/illness	No	559	96.4
	Yes	260	1.4
	I do not know	18	2.2
Signs and symptoms	Diarrhoea	14	1.7
	Vomiting	9	1.1
	Coughing	210	25.6
	Blood in stool	20	2.4
	Allergy	7	0.9
Had breakfast	Yes	96	11.9
	No	710	88.1
Number of meals per day	1	33	4.1
	2	610	75.8
	3	149	18.5
	4–5	13	1.6
Household dietary diversity score	Low	132	14.3
	Medium	652	70.5
	High	141	15.2

**Table 4:** Univariate analysis of factors associated with stunting, underweight, and wasting among schoolchildren in Ifakara, Tanzania, in 2019.

Variable	Category	Univariate Analysis											
		Stunted (HAZ < −2 SD)				Underweight (WAZ < −2 SD)				Wasting (BAZ < −2 SD)			
		Yes	No	OR [95% CI]	*p*-Value	Yes	No	OR [95% CI]	*p*-Value	Yes	No	OR [95% CI]	*p*-Value
Child’s sex	Female	57	444	Ref.		15	362	Ref.		19	406	Ref.	
	Male	53	376	0.91 [061–1.35]	0.63	13	317	1.01 [0.47–2.15]	0.90	22	483	0.73 [0.38–1.37]	0.32
Child’s age	6–8 years	32	484	Ref.		10	506	Ref.		15	501	Ref.	
	9–12 years	78	336	3.51 [2.27–5.42]	0.00	18	176	5.17 [2.34–11.42]	0.00	27	387	2.40 [1.24–4.66]	0.00
Malaria	Negative	94	697	Ref.		20	580	Ref.		32	759	Ref.	
	Positive	2	10	1.48 [0.32–6.87	0.61	0	7	0.00 [0.00–inf]	1.00	0	12	0.00 [0.00–inf]	1.00
Meals per day	1	4	29	Ref.		1	28	Ref.		0	32	Ref.	
	2	79	531	1.08 [0.37–3.15]	0.89	15	447	0.93 [0.12–7.37]	0.95	29	581	1.54 [0.20–11.69]	0.67
	3	14	148	0.68 [0.21–2.23]	0.53	4	114	0.91 [0.10–8.46]	0.93	5	144	1.02 [0.11–9.08]	0.98
Breakfast	No	91	619	Ref.		17	531	Ref.		32	678	Ref.	
	Yes	6	90	0.45 [0.19–1.07]	0.07	3	68	1.38 [0.39–4.82]	0.61	0	96	0.98 [0.34–2.85]	0.97
Dietary diversity	High	7	134	Ref.		4	118	Ref.		5	136	Ref.	
	Medium	79	573	2.63 [1.19–5.85]	0.02	18	467	1.14 [0.37–3.42]	0.82	30	622	1.22 [0.46–3.23]	0.68
	Low	22	110	3.82 [1.57–9.29]	0.00	6	94	1.88 [0.52–6.87]	0.34	7	125	1.52 [0.47–4.92]	0.49
Caregiver’s age	18–35 years	39	330	Ref.		13	273	Ref.		14	355	Ref.	
	36–45 years	26	177	1.24 [0.73–2.11]	0.42	5	149	0.70 [0.25–2.01]	0.51	11	192	1.42 [0.61–3.30]	0.41
	>45 years	22	129	1.44 [0.82–2.52]	0.19	3	105	0.60 [0.16–2.15]	0.41	7	144	1.32 [0.52–3.39]	0.55
Caregiver’s sex	Female	61	484	Ref.		18	390	Ref.		25	520	Ref.	
	Male	26	154	0.75 [0.45–1.22]	0.24	3	138	2.12 [0.61–7.32]	0.23	7	173	1.09 [0.46–2.59]	0.84
SES	Least poor	34	224	Ref.		2	187	Ref.		10	248	Ref.	
	Middle	20	226	0.70 [0.37–1.31]	0.27	11	180	1.34 [0.51–3.55]	0.55	12	234	0.92 [0.38–2.20]	0.84
	Poor	33	188	1.40 [0.78–2.46]	0.26	8	161	1.09 [0.38–3.08]	0.87	10	211	0.92 [0.37–2.27]	0.86
Number of people living in the same household	2–3	10	97	Ref.		12	314	Ref.		22	400	Ref.	
	4–5	54	387	0.76 [0.39–1.47]	0.42	10	167	0.53 [0.18–1.58]	0.26	11	226	1.28 [0.48–3.42]	0.62
	≥6	44	332	1.16 [0.61–2.22]	0.65	3	46	0.69 [0.23–2.06]	0.51	2	63	0.44 [0.14–1.38]	0.16
Anaemia	Normal	85	627	Ref.		17	695	Ref.		25	685	Ref.	
	Mild	4	43	0.68 [0.24–1.95]	0.48	1	46	1.10 [0.14–8.60]	0.92	1	46	0.59 [0.07–4.49]	0.61
	Moderate	7	32	1.61 [0.69–3.77]	0.27	2	37	1.99 [0.44–9.03]	0.37	3	36	2.28 [0.66–7.91]	0.19
	Severe	0	3	3.48 [0.00–inf]	0.98	0	3	1.46 [0.00–inf]	0.98	1	2	13.7 [1.20–156.14]	0.03

BAZ = body mass index—for age z-score; CI = confidence interval; HAZ = height—for-age z-score; OR = odds ratio; SD = standard deviation; WAZ = weight—for-age z-score.

**Table 5:** Factors associated with stunting, being underweight and wasting among schoolchildren in Ifakara, Tanzania, in 2019, according to a multivariate analysis.

Variable	Category	Multivariate Analysis											
		Stunted (HAZ < −2 SD)				Underweight (WAZ < −2 SD)				Wasting (BAZ < −2 SD)			
		Yes	No	OR [95% CI]	*p*-Value	Yes	No	OR [95% CI]	*p*-Value	Yes	No	OR [95% CI]	*p*-Value
Child’s sex	Female	57	444	Ref.		15	362	Ref.		19	406	Ref.	
	Male	53	376	0.89 [0.54–1.46]	0.64	13	317	1.41 [0.46–4.30]	0.54	22	483	1.08 [0.45–2.57]	0.85
Child’s age	6–8 years	32	484	Ref.		10	506	Ref.		15	501	Ref.	
	9–12 years	78	336	3.48 [2.02–5.99]	0.00	18	176	3.86 [1.26–11.77]	0.01	27	387	2.77 [1.11–6.89]	0.02
Malaria	Negative	94	697	Ref.		20	580	Ref.		32	759	Ref.	
	Positive	2	10	2.48 [0.40–15.34]	0.32	0	7	0.00 [0.00–inf]	0.99	0	12	1.90 [0.00–inf]	0.99
Meals per day	1	4	29	Ref.		1	28	Ref.		0	32	Ref.	
	2	79	531	1.09 [0.29–4.04]	0.88	15	447	2.88 [0.00–inf]	0.99	29	581	4.62 [0.00–inf]	0.99
	3	14	148	0.77 [0.18–3.20]	0.72	4	114	4.55 [0.00–inf	0.99	5	144	2.34 [0.00–inf]	0.99
Breakfast	No	91	619	Ref.		17	531	Ref.		32	678	Ref.	
	Yes	6	90	0.47 [0.16–1.38]	0.17	3	68	1.18 [0.22–6.17]	0.84	0	96	1.64 [0.43–6.28]	0.46
Dietary diversity	High	7	134	Ref.		4	118	Ref.		5	136	Ref.	
	Medium	79	573	1.66 [0.71–3.88]	0.24	18	467	0.98 [0.24–4.00]	0.97	30	622	0.62 [0.19–1.99]	0.42
	Low	22	110	2.79 [1.05–7.47]	0.02	6	94	1.69 [0.29–9.96]	0.55	7	125	1.03 [0.24–4.41]	0.96
Caregiver’s age	18–35 years	39	330	Ref.		13	273	Ref.		14	355	Ref.	
	36–45 years	26	177	1.11 [0.62–2.02]	0.73	5	149	0.78 [0.22–2.73]	0.69	11	192	1.22 [0.45–3.37]	0.68
	>45 years	22	129	1.16 [0.60–2.25]	0.65	3	105	0.70 [0.14–3.52]	0.67	7	144	1.42 [0.44–4.50]	0.55
Caregiver’s sex	Female	61	484	Ref.		18	390	Ref.		25	520	Ref.	
	Male	26	154	0.86 [0.49–1.54]	0.62	3	138	2.63 [0.51–13.51]	0.24	7	173	1.46 [0.49–4.30]	0.48
SES	Least poor	34	224	Ref.		2	187	Ref.		10	248	Ref.	
	Middle	20	226	0.53 [0.27–1.01]	0.05	11	180	2.88 [0.56–14.82]	0.20	12	234	0.78 [0.26–2.36]	0.67
	Poor	33	188	0.93 [0.52–1.69]	0.82	8	161	2.67 [0.47–15.07]	0.26	10	211	1.09 [0.38–3.11]	0.85
Number of people living in the same household	2–3	10	97	Ref.		12	314	Ref.		22	400	Ref.	
	4–5	54	387	0.54 [0.24–1.22]	0.14	10	167	0.39 [0.08–1.33]	0.12	11	226	1.16 [0.31–4.30]	0.81
	≥6	44	332	0.85 [0.38–1.90]	0.70	3	46	0.30 [0.07–1.30]	0.11	2	63	0.37 [0.08–1.67]	0.19
Anaemia	Normal	85	627	Ref.		17	695	Ref.		25	685	Ref.	
	Mild	4	43	0.70 [0.23–2.16]	0.54	1	46	0.96 [0.09–9.80]	0.97	1	46	0.67 [0.08– 5.48]	0.71
	Moderate	7	32	2.69 [0.96–7.50]	0.05	2	37	1.96 [0.21–18.32]	0.55	3	36	2.87 [0.59–14.04]	0.19
	Severe	0	3	0.00 [0.00–inf]	0.98	0	3	0.00 [0.00–inf]	0.99	1	2	1.07 [0.78–146.30]	0.07

## Data Availability

All the data for this study will be available upon reasonable request.
